# Gastric duplication cyst with a red flag presentation: a case report

**DOI:** 10.11604/pamj.2021.39.141.29895

**Published:** 2021-06-18

**Authors:** Dinesh Alagoo, Harivinthan Sellappan, Jayaprihyan Jayasilan, Nornazirah Azizan, Firdaus Hayati

**Affiliations:** 1 Department of Surgery, Queen Elizabeth Hospital, Ministry of Health Malaysia, Kota Kinabalu, Sabah, Malaysia,; 2 Department of Pathology and Microbiology, Faculty of Medicine and Health Sciences, Universiti Malaysia Sabah, Kota Kinabalu, Sabah, Malaysia,; 3 Department of Surgery, Faculty of Medicine and Health Sciences, Universiti Malaysia Sabah, Kota Kinabalu, Sabah, Malaysia

**Keywords:** Duplication cysts, gastrointestinal bleeding, hypergastrinaemia, case report

## Abstract

Gastric duplication cyst (GDC) is a rare congenital malformation of the gastrointestinal (GI) tract. Despite being benign in the entity, its complications vary from an asymptomatic abdominal mass to fulminant or massive GI bleeding. A 28-year-old lady presented with unexplained GI haemorrhage, in which the upper endoscopy showed a classic feature of GI stromal tumour. The preoperative diagnosis was also confirmed by the computed tomography. She subsequently underwent surgical resection and the final histopathology was consistent with a benign entity of GDC.

## Introduction

Gastric duplication cyst (GDC), a rare congenital malformation, is typically associated with aberrations identified along the epithelial lining and through attachments to the alimentary tract. Frequently found in the ileum, oesophagus, and colon, while rarely present in the pharynx and tongue, duplication cyst is capable of occurring anywhere within the gastrointestinal (GI) tract [1]. Gastric duplication cyst comprises 4% of all GI duplications [2]. In close to half of the patients, congenital irregularities including alimentary tract duplications, oesophageal diverticulum, or spinal cord abnormalities were often detected [3]. The complications of GDC vary from an asymptomatic abdominal mass to fulminant or massive GI bleeding, and are most frequently diagnosed in young children. We present a 28-year-old lady, who was suspected to have a GI stromal tumour (GIST) through endoscopy but the final histopathology was consistent with a benign entity of GDC.

## Patient and observation

**Figure 1:**
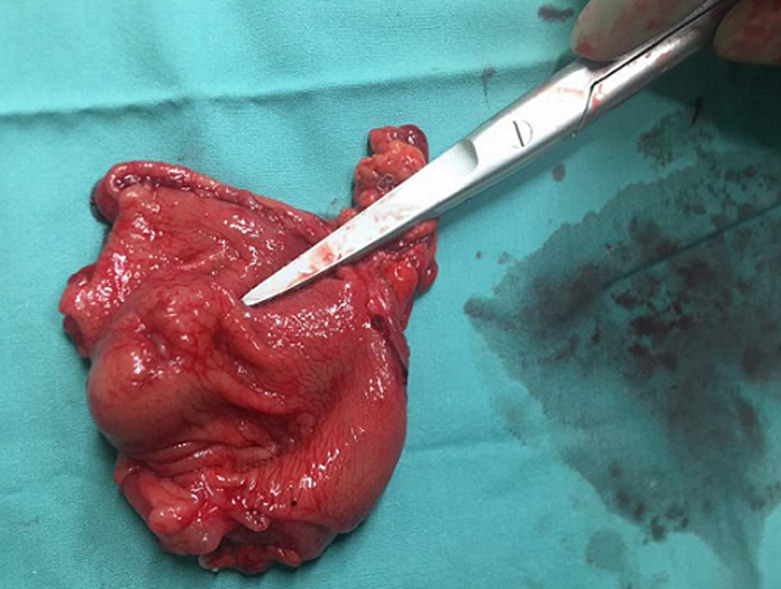
the gross appearance of the specimen showing that the tumor arises from the submucosal layer and appears to have a central umbilication

**Figure 2:**
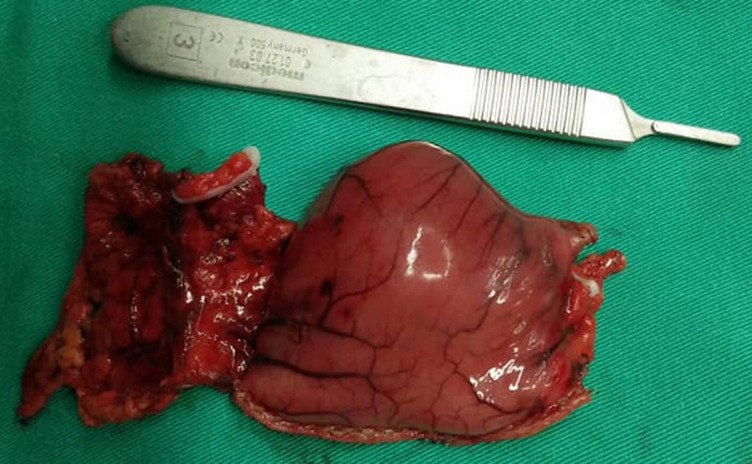
the serosal surface of the specimen showing no tumour breach

**Figure 3:**
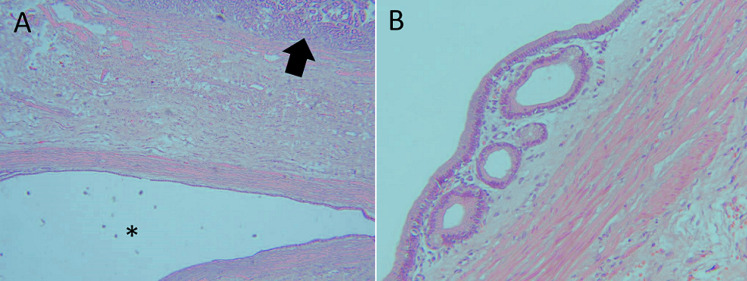
A) the histologic section denoting a surface foveolar epithelium of the gastric antral type mucosa (arrow) with underlying submucosal cyst (*); (haematoxylin and eosin stain x4 magnification); B) another section showing multiple cystically dilated glands lined by gastric foveolar epithelium with higher magnification highlighting a single layer of cuboidal epithelium of the cyst with no nuclear atypia; (haematoxylin and eosin stain x20 magnification)

A 28-year-old lady, presented with a symptomatic anaemia due to an unexplained GI haemorrhage. She had two visits to the emergency department due to recurrent anaemic symptoms prior to her admission. She denied traditional medication usage or family history of malignancy. Clinically she was anaemic but not jaundiced. Examination of the abdomen revealed no mass. Her haemoglobin levels ranged between 8-9 g/dL (normal value: 10-12). She was then transfused with one pint of pack cells blood and was planned for an upper GI endoscopy. The oesophago-gastro-duodenoscopy was performed and showed a submucosal polypoidal mass at the greater curvature with a central umbilication that represents a GIST. There was no active bleeding from the lesion. Even though there was no other source of intragastric pathology, it was concluded that the anaemia was due to a bleeding GIST. This was solidified based on her computed tomography (CT) given that it was a pyloric submucosal lesion with no evidence of metastatic lesions. She was then electively subjected for a laparoscopy-assisted antrectomy and Roux-en-Y reconstruction. The surgery was uneventful. The gross specimen revealed to be a submucosal lesion with a central umbilication (Figure 1, Figure 2). There were no necrotic, haemorhagic or cystic areas visualized. The final histopathology, however, revealed to be a benign entity of GDC (Figure 3). Her colonoscopy then showed to have multiple sigmoid polyps, which turned out to be hyperplastic polyps. She is now on yearly follow up.

## Discussion

GDC is a peculiar congenital malformation. Though the pathophysiology of the congenital development is unknown, several mechanisms have been proposed including recanalization failure of the bowel lumen following the solid-epithelial phase of the intestinal development, persistence of epithelial outpunching in intestine, intestinal ischemia in early intrauterine life, and so on [4]. In majority, it is spherical non-communicating cysts. It rarely interacts with the chest wall, spleen, pancreatic duct, and the intrahepatic bile duct [5]. As in our case, it is a communicating type involving the stomach. What we noted in our case was in agreement with previous case reports where it was predominantly found in the greater or lesser curvature of the stomach [5].

In a majority of the patients, GDC is often detected in the first year. It is only diagnosed in adults upon an incidental finding or due to a complication [6]. The gastric outlet obstruction often leads to abdominal mass or vomiting and occasionally gastric ulcer, perforation, haemorrhage, abdominal pain, hemoptysis, pancreatitis, or hemobilia [6]. Based on past studies, GDC-associated massive GI haemorrhage was rarely detected. However, it is crucial to note that GDC is associated with different types of ulcers, including gastric, duodenal, or colonic, which cause internal bleeding, perforation, abdominal pain and penetration. In our case, we concluded that the source of anaemia is due to bleeding GDC as the endoscopy and radiologic investigations had ruled out other pathologies [7]. An important ulcer-precipitating factor, hypergastrinemia, is a unique association with GDC. However, serum gastrin levels were not measured in our patient.

In managing anaemia secondary to an occult bleeding, the sources of the bleeder must be identified. The initial step is to do an upper and followed by a lower endoscopy as it may be from the upper GI pathology [8]. If the bleeder arises from any gastric pathology as in this case, a complete surgical resection is the best treatment modality. By complete resection, it can lead to resolution of symptoms and elimination of potential malignancy [9]. Based on the endoscopic view, our patient showed a classic presentation of a GIST, hence it required a complete resection. Compared to the open approach, opting for a minimally invasive procedure allows for minimal stay in the hospital and lesser complications, while maintaining patient’s safety and efficacy.

## Conclusion

GDC can manifest as a massive GI haemorrhage. It also can mimic endoscopic features of a GIST. Surgery is mandatory as it is symptomatic with histologic dilemma. Wherever possible, minimally invasive surgery is advisable.
